# Allergic Contact Cell-Mediated Hypersensitivity in Psoriasis: A Narrative Minireview

**DOI:** 10.3390/medicina58070914

**Published:** 2022-07-09

**Authors:** Ana Maria Alexandra Stănescu, Ana-Maria-Antoaneta Cristea, Gabriel Cristian Bejan, Mariana Vieru, Anca Angela Simionescu, Florin-Dan Popescu

**Affiliations:** 1 Department of Family Medicine, Carol Davila University of Medicine and Pharmacy, 050474 Bucharest, Romania; alexandrazotta@yahoo.com; 2 Department of Allergology and Clinical Immunology, Nicolae Malaxa Clinical Hospital, 022441 Bucharest, Romania; ana.savoiu@gmail.com (A.-M.-A.C.); florindanpopescu@gmail.com (F.-D.P.); 3 Department of Allergology, Carol Davila University of Medicine and Pharmacy, 050474 Bucharest, Romania; 4 Department of Obstetrics and Gynecology, Carol Davila University of Medicine and Pharmacy, 050474 Bucharest, Romania; asimion2002@yahoo.com; 5 Department of Obstetrics and Gynecology, Filantropia Clinical Hospital, 011132 Bucharest, Romania

**Keywords:** psoriasis, contact hypersensitivity, patch test

## Abstract

The dysfunctionality of the protective skin barrier in psoriasis allows easier cutaneous penetration of various contact haptens; thus, such patients can develop allergic contact hypersensitivity as a comorbidity. Both skin conditions involve T-cell-mediated mechanisms. Dermatologists and allergists should consider assessing allergic contact cell-mediated hypersensitivity in selected psoriasis patients, especially those with palmoplantar psoriasis and who are refractory to topical treatments, and in patients with psoriasis, with or without arthritis, treated with biologics that present skin lesions clinically suggestive of contact dermatitis.

## 1. Introduction

Psoriasis and allergic contact dermatitis are common inflammatory skin disorders, characterized by epithelial alterations and a T-cell-mediated immunopathology, involving either autoantigens or allergens. Psoriasis is characterized by a hyperproliferation of keratinocytes and a massive skin infiltration of immune cells. It affects all age groups, and its prevalence in the general population varies from 1% to 3%, with differences in various geographical areas due to environmental and genetic factors. Allergic contact dermatitis is caused by contact cell-mediated hypersensitivity and has a prevalence of about 15–20% in the general population [1,2,3]. This type IV hypersensitivity may be assessed in vivo with skin patch testing in selected patients with psoriasis and may be treated with either refractory or topical treatments, or with biologics if the patient is presenting with cutaneous lesions clinically suggestive of contact dermatitis. There are not many published papers assessing the relationship between psoriasis and allergic contact dermatitis or contact cell-mediated hypersensitivity. 

## 2. Psoriasis and Allergic Contact Hypersensitivity as T-Cell-Mediated Conditions

The integumentary system is the largest organ in the human body, acting as a physical barrier between external and internal environments, and participates in regulating the hydro-electrolytic balance, body temperature, the synthesis of vitamin D, the detection of stimuli, and innate immune mechanisms. Human skin consists of three layers: the epidermis, dermis, and hypodermis, and skin appendages, and can be affected by a multitude of diseases, including inflammatory conditions such as psoriasis [4]. The different levels of the cutaneous barrier are: the physical, chemical, microbiome, and immune barriers, with a highly interconnected network of cells and mediators [5].

Psoriasis is a common immune-mediated inflammatory skin disease, with a chronic relapsing–remitting course, characterized by an abnormal proliferation/differentiation of keratinocytes and excessive immune cell infiltration in the epidermis and dermis [6,7].

Environmental stress and trauma may act as possible triggers of psoriasis, particularly in subjects with genetic susceptibilities, such as the psoriasis-susceptibility locus *PSORS1*. This locus induces the release of the chemokines CCL20, CXCL8, and CXCL1, stimulating CCR6+ IL-17+ lymphocytes, neutrophils, and IL-23+ CD14+ dendritic cells, respectively. The release of the cytokines IL-17A and IL-17F by Th_17_ and Tc_17_ lymphocytes, and by innate lymphoid cells ILC3, results in the activation of the IL-17RA/IL-17RC complex in keratinocytes, further amplifying the inflammatory response. Moreover, the hyperproliferation of keratinocytes is accelerated by the production of IL-22. Dendritic cells induce cytokines such as IL-1*β*, TNF-*α*, and IL-23. IL-17-producing cells are stimulated by IL-23 via the JAK2/TYK2-STAT3 pathway [8]. Epidermal Langerhans cells have an altered migratory capacity to drain lymph nodes, and consequently, there is a delayed onset of skin immune responses. The capacity of the structural keratinocytes to produce a diversity of cytokines and chemokines, along with the hyperproliferation process, lead to a vicious pathogenesis cycle. Proinflammatory Th_1_ and Th_17_ cells dominate the affected skin, producing changes in the microbiome composition and production of antimicrobial peptides [9].

Some genes involved in the skin barrier function are linked to psoriasis. In the psoriasis susceptibility *PSORS4* region, more than 60 genes that control keratinocyte differentiation are encoded [10]. The deletion of *LCE3B* and *LCE3C* genes, responsible for encoding late cornified envelope proteins, is associated with psoriasis [11]. Regarding antimicrobial late cornified envelope proteins, the psoriasis-risk-factor deletion of *LCE3B/C* genes also affects microbiota composition [12].

Because psoriasis alters the skin’s protective barrier, different microbes, irritants, and contact haptens/allergens more easily penetrate the cutaneous layer with presentation to dendritic cells. Therefore, allergic contact hypersensitivity has been reported as a possible comorbidity in psoriasis patients [1,2,3,13].

Allergic contact cell-mediated hypersensitivity involves epithelial alterations and multifaceted T-cell-mediated mechanisms, and is the critical mechanism involved in allergic contact dermatitis, a frequent inflammatory skin disorder triggered by epicutaneous contact with haptens in sensitized people [1,14,15,16,17,18]. Contact hypersensitivity has been reported to occur more frequently in women, with a ratio of women to men of 2:1, likely due to social, behavioral, and environmental factors [16,17,18].

Psoriasis lesions are clinically described as raised red plaques covered by white scales, well-demarcated and variable in size. Under the white scales, homogeneous erythema and bleeding points can be detected when the lesions are traumatized and the scales are scraped off; this is a diagnosis marker known as the Auspitz sign. Usually, the distribution of lesions is symmetric. Allergic contact dermatitis lesions vary depending on whether the stage is acute or chronic, and on the severity. The pruritic lesions are characterized first by erythema, edema, papules, vesicles, and, over time, scaling and lichenification [15,19].

According to the histological assessment of psoriasis samples, the main features of psoriasis are epidermal hyperplasia with the elongation of rete ridges, parakeratosis associated with focal orthokeratosis and the presence of neutrophil (Munro) microabscesses, the disappearance of the granular layer, and T-cell infiltrate. Typically, allergic contact dermatitis lesions reveal an infiltration of T cells in the upper dermis, with spongiosis primarily in the lower epidermis, and keratinocyte apoptosis induced by T cells. Apoptotic processes, a hallmark of allergic contact dermatitis, are opposed to psoriasis histopathology, characterized by an apoptosis-resistant and metabolic-active epidermis [15,16,19].

In the last decade, published data have led to advancements in understanding the pathogenesis of psoriasis and allergic contact hypersensitivity. After years of research, both conditions are now considered T-cell-mediated skin disorders involving multilayered innate and adaptive immunity mechanisms [15,20].

Initially, psoriasis was considered a Th_1_-mediated inflammatory disease, especially with the involvement of interferon IFN-*γ*, along with the contribution of tumor necrosis factor TNF-*α* and interleukin IL-2. IFN-*γ*. TNF-*α* stimulates keratinocytes to synthesize cytokines such as IL-6 and IL-8, and it is responsible for the secretion of additional inflammatory mediators by increasing intercellular adhesion molecule ICAM-1 expression [20]. Later, the study of immune mechanisms focused on pathogenetic T cells, represented not only by Th_1_ lymphocytes, but also Th_17_ cells [11,12], as studies showed that the CD4+ and CD8+ T cells from psoriasis lesions express IL-17, which is involved in the amplification of the neutrophil chemotaxis induction of microabscesses [12]. Although traditionally, research has focused on the role of αβ T cells, it has become increasingly evident that *γ*δ T cells, particularly those that are IL-17A-producing, contribute to inflammatory skin diseases, such as psoriasis and allergic contact dermatitis [1]. In addition to the significant contribution of IL-17 to the immunopathogenesis of psoriasis, keratinocytes, macrophages, dermal dendritic cells, and Langerhans cells produce large amounts of IL-23. This cytokine stimulates certain subsets of CD4+ T cells that induce the expressions of IL-17 and IL-22. This process plays a key role in the pathogenesis mechanism that maintains the inflammation in psoriasis [19,21].

Allergic contact dermatitis is based on a cell-mediated hypersensitivity reaction with an initial sensitization phase when the patient does not present with well-defined clinical symptoms, followed by the elicitation phase, which is associated with defined skin lesions [21,22]. At first, this hypersensitivity reaction was described as a Th_1_-mediated chronic inflammatory skin condition. During the first phase, contact with haptens is followed by the local release of TNF-*α*. The elicitation phase involves interferon IFN-*γ*, but also increased IL-6 and IL-8 levels [20,21]. In the last few years, some studies have reported a significant proportion of Th_17_ cells. The secreted levels of IL-17 by CD4+ T-cell clones may play a significant role in amplifying local inflammation, because the keratinocytes are stimulated to synthesize proinflammatory cytokines [20,21,23].

## 3. Skin Patch Testing for Contact Cell-Mediated Hypersensitivity in Psoriasis

The association between psoriasis and allergic contact hypersensitivity is still a point of discussion and an area for future research. In psoriasis patients, the coexistence of contact hypersensitivity may explain some lesions’ locations, treatment resistance, and prognosis with increased skin disease morbidity [2,24,25]. Some authors reported the precipitation of the Köbner phenomenon due to allergic contact hypersensitivity. They suggested investigating a possible contact sensitization if the pruritic skin lesions involve pustules and vesicles [26].

Patch testing is the gold-standard diagnostic method to detect allergic contact cell-mediated hypersensitivity, but expertise is required to perform and interpret patch tests and to properly assess particularities of specific cases [27,28,29,30].

Several studies have revealed higher incidence rates of contact hypersensitivity reactions in patients with psoriasis, while others have shown an inverse relationship between the two skin conditions, and that both conditions have the same prevalence in healthy subjects [2,22,25,31,32,33].

The first research paper that noted a significant association between psoriasis and allergic contact hypersensitivity was published in 1998 by Heule et al. These researchers reported a high percentage of 68% positive patch tests; however, the studied group included only 47 patients [34].

The North American Contact Dermatitis Group evaluated 38,723 patients patch-tested between 2001 and 2016, 1675 of whom had psoriasis. The results showed that 32.7% of psoriasis patients had positive patch tests, while 57.8% of nonpsoriasis patients had positive tests [33].

Claßen et al. recently investigated a group of 2294 patients with psoriasis and found 25% of subjects with at least one positive skin patch test versus the control group, which presented 39% positive subjects. Regarding the proportion of positive patients, the authors considered that almost 60% of psoriasis-positive individuals were tested, excluding sensitization, because the physicians did not expect contact hypersensitivity [32].

Some papers have reported a difference between the identified positive patch tests in female psoriasis patients compared with male patients, which is likely because women are more frequently exposed to various accessories, jewelry, and cosmetic products with fragrances and other sensitizing haptens [22,35]. Jovanović et al. found that 27.7% of female patients were sensitized to contact haptens compared with only 5.8% of male patients. Another finding was the significantly lower percent of sensitized male patients versus healthy tested male subjects (24.1%) [35]. In contrast to the findings of Jovanović et al., Heule et al. found a similar prevalence of affected men and women: 66.7% and 68.3%, respectively [34].

Psoriasis personal history may also play a role in the association with allergic contact hypersensitivity. The sensitization rate is higher in patients with a disease duration of at least five years. This can be explained by the ongoing use of numerous topical treatments and personal care products, such as emollient creams, shampoos, and ointments [31,36].

A more frequent association between psoriasis and allergic contact hypersensitivity was seen in patients with palmoplantar psoriasis. The exposure of palms and soles with an altered protective skin barrier to various common sensitizers and different topical treatments increased the sensitization risk [22]. By skin patch-testing palmoplantar psoriasis patients, Žužul et al. found that 29.5% were sensitized [3], while Lipozencić et al. reported 41.7% positive patients, compared with only about 6.6% allergic patients in subjects with other psoriasis clinical phenotypes [37]. Therefore, it was proposed to perform patch tests in patients with palmoplantar psoriasis who are refractory to treatment [2,22].

A significant topic of research has been the most common sensitizing contact allergens in psoriasis patients. Alwan et al. reported that the involved haptens are similar to those in nonpsoriasis subjects. The non-noble metal nickel was the most frequently reported (17.6%), followed by a fragrance mix containing amyl cinnamal, cinnamal, hydroxycitronellal, cinnamyl alcohol, eugenol, geraniol, isoeugenol, and oakmoss absolute (7%); the biocide methylisothiazolinone (6.1%); the transition metal cobalt (5.3%); and the natural resin colophonium (4.2%). Among the top ten haptens, only paraphenylenediamine, a primary intermediate commonly used in oxidative hair dyes, had a lower frequency in the psoriasis group (2.7%) than in the nonpsoriasis one (3.6%) [38]. Likewise, García-Souto et al. found the most frequent sensitizing hapten in a psoriasis group to be the metal hapten nickel sulfate (34.6%), followed by the biocide mix methylchloroisothiazolinone/methylisothiazolinone (6.2%), and the organomercurial preservative thimerosal, also known as thiomersal (5.4%), comparable to the frequency of contact allergens within an atopic dermatitis group [2]. Other publications have reported the precious metal salt sodium gold thiosulfate, Peru balsam resin, and the antibiotics neomycin and bacitracin to be common sensitizing allergens [3,32,33]. In palmoplantar psoriasis, Žužul et al. reported the transition metal salts potassium dichromate and cobalt chloride; thiomersal preservative; the rubber additive carba mix, classically containing diphenylguanidine, zinc diethyl- and dibutyl-dithiocarbamate; and the topical ester local anesthetic benzocaine to be frequent sensitizers [3].

Avoiding cutaneous contact with all these hapten triggers leads to the clinical improvement in skin lesions, with no additional worsening due to re-exposure [36].

Another factor that influences the prevalence of sensitized patients is related to the applied patch-test series. Skin testing with extended hapten series helps to find more sensitizations [34].

In addition, we discuss contact sensitization to haptens found in topical therapies for psoriasis patients. Coal tar was found to be a relevant hypersensitivity association due to its presence in some shampoos and lotions used in psoriasis treatment. One study reported dithranol to be a contact trigger in 6.5% of patients. These haptens may be involved in cases refractory to treatment [2,26,31,34,38]. A recent case of allergic contact dermatitis in a psoriasis patient caused by the excipient cetylstearyl alcohol was recently reported by Navarro-Triviño et al. [39]. Jovanović et al. investigated the use of herbal topical products by an increasing number of patients, and found that Compositae/Asteraceae contact allergens were common sensitizing factors in psoriasis. However, this issue is likely underreported, perhaps because these patients are not frequently patch-tested [35].

Claβen et al. revealed that, with the exceptions of the rubber additive mercapto mix and the preservative paraben mix, being a psoriasis patient may be associated with a so-called “protective effect” on contact sensitization to the haptens from the European baseline series, independent of the affected body site, sex, or age [32].

García-Souto et al. studied the correlation between allergic contact hypersensitivity and the exposure of psoriasis patients to humidity at their workplace. About 34% of patients from the studied group were cleaners, although the research also included patients with different types of wet-work and non-wet-work jobs. In terms of contact hypersensitivity, these authors emphasized the need for specific preventive measures for workers with psoriasis to avoid continuous skin exposure to water at the workplace [2].

Another significant issue related to allergic contact hypersensitivity in psoriasis is the possible late positivity of patch tests related to this skin disease. Most of the published studies reported the results of patch testing after days three or four, but it was noticed that in some patients, the positive responses developed on day seven or later. Quaranta et al. reported that in a control group, the peak of the patch skin reaction was achieved 3–5 days after hapten application, while in the psoriasis group, the peak of the positive reaction was observed after seven days. A possible explanation for this may be linked to keratinocyte hyperproliferation and the resistance to apoptosis, in contrast to allergic contact dermatitis. An additional issue may have been the altered gene expression involved in the proliferation and metabolic processes of psoriasis [15,33,40]. In brief, the benefit of late readings in patch testing depends, not only on allergens, as previously suggested, but also on patient characteristics [41].

Moreover, the controversial results may have been due to sun exposure, ultraviolet light therapy, among other treatments for psoriasis, such as topical immunomodulators or systemic immunosuppressive drugs, which may have influenced patch testing results [23,31,32]. Consequently, some studies considered such therapies to be exclusion criteria [34].

Furthermore, nail dystrophy mimicking psoriatic nails may be an overlooked clinical presentation of allergic contact dermatitis, caused by different haptens present in nail cosmetics. In the past, tosylamide formaldehyde resin was a common contact allergen in nail varnish, but due to changes in consumers’ exposures, its significance has diminished. Formaldehyde in nail hardeners and methacrylate in acrylic nails may induce nail dystrophy. Several cases of acrylate contact allergies presenting solely with pseudo-psoriatic nails have been reported in the literature, and the lack of typical eczematous changes on skin may have led to misdiagnoses and delays in patch testing with methacrylate [42,43,44,45]. The rate of contact allergies to synthetic trimellitic anhydride copolymers used in nail polish and varnishes has also been relatively high, but these are not available as commercial haptens for patch testing. Testing with individual materials, such as nail varnish “as is” and nail polish top and base coats containing such copolymers, is important to avoid missing relevant contact allergies. In addition, by attempting to hide dystrophic nails, patients may continue to use nail cosmetics, thus unintentionally aggravating the condition [46,47].

The inability to perform patch testing due to the unavailability of patch test kits was recently considered an important limitation when assessing palmar psoriasis and eczema in psoriasis patients [48].

## 4. Psoriasis Biologics and Skin Patch Testing

Furthermore, psoriasis patients may be treated with biological therapies targeting TNF-*α*, IL-17, and IL-23. Because contact cell-mediated allergy and psoriasis are T-cell-mediated, involving Th_1_ and Th_17_ lymphocytes, and TNF-α, IL-17, and IL-23 are cytokine mediators in their pathogeneses, some authors have assessed the skin patch testing in patients with psoriasis treated with biologics. However, such publications in the literature rarely considered adalimumab, etanercept, infliximab, ixekizumab, secukinumab, and ustekinumab [13,49,50,51].

The suppression of delayed contact hypersensitivity was revealed in TNF-*α* gene-deficient mice, with this important proinflammatory cytokine playing an enhancing role in the elicitation phase of such an immune reaction [52].

A first case report presented a patient with psoriasis and psoriatic arthritis with long-term treatment with infliximab (a chimeric mouse–human monoclonal antibody that inhibits TNF-α), who developed chronic allergic contact dermatitis of the hands, confirmed by a skin biopsy and multiple sensitizations to various contact haptens. His occupational exposure involved metals, coolants, oils, and nitrile rubber gloves. Skin patch testing with a reading at 72 h revealed significant positive results for nickel sulfate, formaldehyde, ethylenediamine dihydrochloride, dimethyl oxazolidine, thiocarbamate, and thiuram rubber additives. This successful patch testing revealed that TNF-*α* blockers did not necessarily suppress cell-mediated contact hypersensitivity and did not represent a contraindication to performing skin patch tests [53].

Myers et al. published another case report of acute contact dermatitis, presumably caused by Rhus urushiol exposure, in a patient with psoriasis and psoriatic arthritis treated with etanercept (a soluble recombinant human TNF-*α* inhibitor), suggesting that cutaneous delayed-type hypersensitivity reactions may not be impaired by this biological therapy [54].

Wee et al. assessed several patients with psoriasis treated with etanercept, infliximab, adalimumab, methotrexate, mycophenolate, and ciclosporin, and found positive patch test results for various epicutaneously applied haptens in all of them, suggesting that if immunosuppressive agents cannot be stopped, then patch testing with a careful reading of the results may be performed in such patients [55].

Kim et al. reported that two of three patients with psoriasis treated with adalimumab (a fully human recombinant anti-TNF-*α* monoclonal antibody) had at least one significant positive patch test reaction, with the haptens mentioned being Peru balsam, methyldibromo glutaronitrile, cobalt chloride, and gold sodium thiosulfate [13].

There is some scientific evidence that the blockage of IL-12/IL-23 may have an impact on the elicitation of allergic contact dermatitis. The keratinocytes of nickel-allergic patients, producing IL-23 in response to nickel stimulation and Th_17_ memory T cells, increase in the peripheral blood of subjects with nickel allergies after nickel exposure. Therefore, the effect of IL-12/IL-23 antagonism on skin patch testing was discussed [27]. The case of a patient with psoriasis and psoriatic arthritis who developed a severe maculopapular exanthem induced by hydroxyzine was published. Positive patch testing to this antihistamine was reported when treated with ustekinumab (a human IgG1*κ* monoclonal antibody directed against the p40 subunit common to IL-12 and IL-23 cytokines) for psoriasis, revealing that this biologic efficiency in psoriasis did not prevent a positive patch test reaction to hydroxyzine [56].

In addition, Bangsgaard et al. treated five patients who had long-standing chronic allergic contact dermatitis with ustekinumab, with positive patch test reactions to more than three contact haptens and reported that only one patient presented with significant clinical improvement, while the other two developed herpes zoster and a serious flare of eczema with *Staphylococcus aureus* and *Streptococcus pyogenes* concomitant infections, respectively. Therefore, this anti-IL-12/23 p40 monoclonal antibody did not appear to be a valuable therapy for chronic allergic contact dermatitis [57].

Hamann et al. reported a psoriasis patient in treatment with secukinumab (a human IgG1*κ* monoclonal antibody that binds to IL-17A) who developed pruritic dermatitis on the hands, arms, legs, and trunk and presented with a positive patch test to the emulsifier sorbitan sesquioleate, and the reactions to fragrance mix I and cinnamic aldehyde were deemed to be attributable to its presence in these patch test products. Because sorbitan sesquioleate was found to be an ingredient in the patient’s clobetasol cream, its avoidance significantly improved the condition [50].

Other researchers have reported a suppression of allergic contact responses in mice with IL-17 deficiencies [50,58]. Wu et al. recently published the off-label effects of secukinumab in a few patients with allergic contact dermatitis, but a minimal improvement was recorded in only one of four [59]. Todberg et al. also presented three patients with allergic contact dermatitis in treatment with secukinumab, but only one experienced a relief of eczema evolution by the end of the treatment [60]. Moreover, in a study of ten patients with nickel contact allergies, secukinumab resulted in a slight decrease in the clinical scoring of nickel patch test reactions but not in inflammation or skin thickness, indicating that anti-IL-17 may not be a viable treatment for allergic contact dermatitis [61].

Because IL-17, elevated in allergic contact dermatitis, has a stimulatory role in the sensitization and effector stages of contact hypersensitivity, and sensitization with contact haptens induces the circulation of Th_17_ memory cells and long-lasting local memory responses [58,62,63,64,65], we discuss the influence of an agent that blocks the inflammatory protein IL-17A in skin patch testing. A patient with plaque psoriasis, without arthritis, and with a favorable response to ixekizumab (a humanized IgG_4_ monoclonal antibody binding to IL17A and blocking the interaction with its receptor, IL-17RA) was reported to have presented with airborne contact dermatitis, related to sleeping in a freshly painted house. Isothiazolinones in the paint were suspected as the cause, and the skin patch tests were positive for methylisothiazolinone and methylchloroisothiazolinone/methylisothiazolinone. The findings of [49] and a study assessing the effect of treatment with anti-IL-17 in patients with allergic contact dermatitis [61] were preliminary and indicated more complex mechanisms in the immunoregulation of allergic contact dermatitis [49]. Further research on the benefit of other biologic treatments in allergic contact dermatitis is needed.

Dupilumab, a fully human monoclonal antibody that binds IL-4R*α* and inhibits the signaling of both IL-4 and IL-13, has been prescribed for the treatment of adult patients with moderate-to-severe atopic dermatitis. Although it has not been indicated for the treatment of psoriasis, this monoclonal antibody may be associated with psoriasis or psoriasiform dermatitis in patients with atopic dermatitis. This type of psoriasis appears to be immunologically distinct from classical psoriasis, and some cases were described as ‘‘psoriasiform’’ because they did not necessarily meet the definition of psoriasis [66,67,68,69,70]. Although a switch in the polarization of the predominant immune response from Th_2_ to Th_17_ was hypothesized, a deeper understanding of the mechanism behind this reaction to dupilumab will lead to the complete comprehension of the immunopathogeneses of psoriasis and atopic dermatitis [71]. A recent systematic review by Mufti et al. assessing studies with dermatitis patients patch-tested before and during various treatments reported that almost 68% of the patients who received dupilumab maintained positive patch testing results to an allergen that was previously graded as a significant positive reaction [72].

Regarding pregnancy and lactation, skin patch testing may be performed if there are urgent reasons for the workup of contact allergies and postponement is not desirable [73], but psoriasis treatment decisions are difficult to make under these conditions [74,75,76]. To the best of our knowledge, there are no reports of positive patch tests in pregnant or lactating women treated with biologics for psoriasis. Because there are no conclusive data in terms of the risks and benefits of skin patch testing under these conditions, as a precaution, patch tests should not be recommended during pregnancy or breastfeeding [29].

## 5. Conclusions

Dermatologists and allergists should consider assessing contact cell-mediated hypersensitivity in selected psoriasis patients, especially those with palmoplantar psoriasis, refractory to topical treatments, and in patients with psoriasis, with or without arthritis, treated with biologics and presenting with skin lesions clinically suggestive of contact dermatitis. Lastly, biologics which significantly inhibit skin patch testing and are efficient in treating the skin disorders psoriasis and allergic contact dermatitis, have currently not been mentioned in the literature.

## Data Availability

Not applicable.
